# One-Pot Anodic Electrodeposition of Dual-Cation-Crosslinked Sodium Alginate/Carboxymethyl Chitosan Interpenetrating Hydrogel with Vessel-Mimetic Heterostructures

**DOI:** 10.3390/jfb16070235

**Published:** 2025-06-26

**Authors:** Xuli Li, Yuqing Qu, Yong Zhang, Pei Chen, Siyu Ding, Miaomiao Nie, Kun Yan, Shefeng Li

**Affiliations:** 1 Hubei Province Key Laboratory of Agricultural Waste Resource Utilization, School of Chemistry and Environmental Engineering, Wuhan Polytechnic University, Wuhan 430023, China; lixuli@whpu.edu.cn (X.L.);; 2 Key Laboratory of Textile Fiber & Product, Ministry of Education, Wuhan Textile University, Wuhan 430200, China

**Keywords:** anodic electrodeposition, interpenetrating networks, cascade reaction, dual-cation-crosslinking, multilayer structure

## Abstract

This study develops a one-pot anodic templating electrodeposition strategy using dual-cation-crosslinking and interpenetrating networks, coupled with pulsed electrical signals, to fabricate a vessel-mimetic multilayered tubular hydrogel. Typically, the anodic electrodeposition is performed in a mixture of sodium alginate (SA) and carboxymethyl chitosan (CMC), with the ethylenediaminetetraacetic acid calcium disodium salt hydrate (EDTA·Na_2_Ca) incorporated to provide a secondary ionic crosslinker (i.e., Ca^2+^) and modulate the cascade reaction diffusion process. The copper wire electrodes serve as templates for electrochemical oxidation and enable a copper ion (i.e., Cu^2+^)-induced tubular hydrogel coating formation, while pulsed electric fields regulate layer-by-layer deposition. The dual-cation-crosslinked interpenetrating hydrogels (CMC/SA-Cu/Ca) exhibit rapid growth rates and tailored mechanical strength, along with excellent antibacterial performance. By integrating the unique pulsed electro-fabrication with biomimetic self-assembly, this study addresses challenges in vessel-mimicking structural complexity and mechanical compatibility. The approach enables scalable production of customizable multilayered hydrogels for artificial vessel grafts, smart wound dressings, and bioengineered organ interfaces, demonstrating broad biomedical potential.

## 1. Introduction

Cardiovascular diseases, as one of the leading causes of global mortality, have driven decades of intensive research into vascular transplantation solutions [1]. While autologous grafts remain the clinical gold standard for severe cases, donor shortages and the high failure rates of synthetic alternatives, particularly for small-diameter vessels (<6 mm), highlight an urgent need for biomimetic substitutes [2,3,4]. Commonly, the native blood vessels achieve exceptional hemodynamic functionality through their hierarchical tri-layer architecture: the endothelial-rich tunica intima regulates thrombosis and inflammation, the smooth muscle-dominated tunica media controls vasomotor activity, and the fibroblast-embedded tunica adventitia provides structural reinforcement [5,6]. Conventional fabrication strategies such as 3D bioprinting [7] and electrospinning [8], though capable of producing tubular constructs, fail to recapitulate this structural hierarchy while maintaining essential biomimetic properties—a limitation rooted in their inability to synchronize spatial organization with dynamic crosslinking kinetics [9,10]. Therefore, the development of rapid, cost-effective methodologies to engineer vessel grafts with tunable internal structure and biological functionality remains a critical unmet need in regenerative medicine [11].

Polysaccharide hydrogels show immense potential as vascular graft materials due to their biomimetic composition, structural adaptability, and tissue-like viscoelastic properties [12,13,14,15]. While these materials can theoretically replicate the architectural complexity of blood vessels through rational design, practical implementation faces three key challenges: selection of biocompatible polymers with appropriate biological activities, precise spatial control over three-dimensional gel architectures, and achievement of mechanical properties matching native vasculature [16,17,18]. In recent decades, the electrophoretic deposition has emerged as a transformative fabrication strategy, overcoming conventional limitations through its spatiotemporal control of gelation kinetics under physiological conditions [19,20]. Unlike UV-curing methods that risk monomer toxicity or diffusion-limited approaches causing structural inhomogeneity [21,22], this technique enables programmable structuring of biopolymers [23,24,25]. The process involves pH-dependent sol-gel transitions and ionic crosslinking mechanisms [26]. Current electrophoretic systems nevertheless face critical constraints, such as the excessive crosslinking density during continuous deposition often yields brittle hydrogels, and the inherent rapid gelation kinetics restrict diffusion-mediated structural refinement. Recent research advances in electrodeposition have achieved the fabrication of multilayered hydrogels with programmable architectures and stimuli-responsive functionalities through synergistic ionic–covalent crosslinking and spatiotemporally controlled deposition, enabling applications in drug delivery, energy storage, and bioelectronics [27,28,29]. Building upon our established electrical methodology for fabricating multilayered chitosan hydrogels [30], this study pioneers a universal pulsed anodic electrodeposition system integrating dual-cation crosslinking and interpenetrating biopolymer networks. To our knowledge, this represents the first demonstration of programmable multilayered hydrogel synthesis through pulsed anodic polarization coupled with cascade diffusion reaction strategies.

Herein, this study develops an electrically programmable strategy for fabricating vessel-mimetic hydrogels through pulsed anodic electrodeposition coupled with wire-templated tubular structure formation. The sodium alginate/carboxymethyl chitosan and ethylenediaminetetraacetic acid calcium disodium salt hydrate (EDTA·Na_2_Ca) have been incorporated to construct a dual-cation-crosslinked interpenetrating hydrogel network. Compared to traditional preparation methods for biomimetic multilayered vascular or wound dressing materials, this study proposes an electrochemically controllable metal-coordinated double-network structure. This structure allows precise regulation of the internal architecture of the multilayered hydrogel via electrical signals, leading to enhanced mechanical properties and controllable interfacial erosion behavior at the multilayer boundaries, thereby achieving controllable release of antibacterial metal ions. The significance of this work lies in its novel concept of transducing electrical signals into complex biological information (e.g., multilayer architecture), serving two key purposes: providing a generic methodology for assembling biopolymer-based multifunctional hydrogels as artificial blood vessels in the short term and bridging the information-processing capabilities of electronics and biology in the long term.

## 2. Materials and Methods

### 2.1. Materials

Chitosan (deacetylation degree of 90%, medium molecular weight of 220 kDa) and ethylenediaminetetraacetic acid calcium disodium salt hydrate (EDTA·Na_2_Ca, ≥97.0%) were received from Sigma-Aldrich (Saint Louis, MO, USA). Sodium alginate (Mw = 6.4 × 10^4^ g/mol, viscosity: 200 ± 20 mPa·s) was received from Sinopharm Chemical Reagent Co. Analytical-grade reagents, including sodium hydroxide pellets (NaOH, ≥99.0%), calcium chloride (CaCl_2_, ≥99.0%), monochloroacetic acid (≥99.0%), and acetic acid (≥99.5%), were utilized without additional treatments.

### 2.2. Preparation of SA/CMC Composite Solution

A sodium alginate (SA) solution was prepared by dissolving 4 g of sodium alginate powder in 100 mL of distilled water under continuous magnetic stirring for 6 h at room temperature. The carboxymethyl chitosan (CMC) solution was synthesized through a modified literature approach [31]: 10 g of chitosan was initially dissolved in 200 mL of 20% NaOH solution with 15 min of magnetic stirring. Subsequently, 30 g of monochloroacetic acid was introduced dropwise at 40 °C over 2 h. The resulting mixture underwent neutralization using 10% acetic acid, followed by filtration, washing with 80% ethanol, and vacuum drying. For solution preparation, 4 g of dried CMC film was dissolved in 100 mL of distilled water through 6 h of magnetic stirring. Equal volumes of SA and CMC solutions were combined and magnetically stirred for 4 h to prepare the SA/CMC mixture (2 wt%/2 wt%, pH~8.5). For comparable study, the SA or CMC solution was provided as the control. When required, 1% EDTANa_2_Ca powder (1 g/100 mL) was incorporated prior to electrodeposition.

### 2.3. Anodic Electrodeposition of SA/CMC-Ca/Cu Tubular Hydrogel

Electrodeposition experiments were conducted using a CHI620E electrochemical analyzer (CH Instruments, Austin, TX, USA) with a copper wire anode (diameter, 1 mm) and Pt wire cathode (diameter, 1 mm). Prior to use, the copper wires were subjected to sequential ultrasonic cleaning in acetone, ethanol, and water (5 min each). Initial trials involved partial immersion (1.5 cm depth) of electrodes in the blend solution under a constant current application for 5 min. Comparative experiments employing alternative polymer solutions were conducted under identical conditions. In some cases, for multilayer hydrogel preparation, a pulsed electrical signal sequence (ON-OFF model) was applied to the electrodes. A typical 3-layer sample can be prepared by processing 3 cycles of ON step (anodic current of 1.5 A/m^2^ for 60 s) and OFF step (anodic current of 0 A/m^2^ for 5 s). After deposition finished, the hydrogel-coated wire was immediately removed from the solution, gently rinsed with water, and then peeled off from the electrode surface for further measurements. For SEM measurements, the deposited hydrogels were rinsed with distilled water, flash-frozen in liquid nitrogen, and freeze-dried at −40 °C for 24 h.

### 2.4. Antibacterial Properties

Antibacterial efficacy against *S. aureus* was evaluated via a modified spread plate protocol. In particular, the as-prepared hybrid hydrogel specimens were co-cultured with a bacterial suspension (bacterial density: 10^8^ CFU/mL) at 37 °C for 1 h. Then, 100 μL of the above-mentioned bacterial suspensions were evenly spread on agar plates using sterile glass beads and co-cultured at 37 °C for 24 h. Colony-forming units (CFUs) were quantified using automated colony counters, and the antibacterial efficiency of the hydrogel was then calculated as

Antibacterial efficiency (%) = (CFUs_control_ − CFUs_sample_)/CFUs_control_ × 100%.

CFUs_sample_ and CFUs_control_ represent the colony-forming unit counts of the suspensions treated with and without hydrogel samples, respectively.

### 2.5. Characterizations

Chemical structures of the hybrid hydrogels were revealed by attenuated total reflection Fourier transform infrared spectrometer (ATR-FTIR, Nicolet 6700, Bruker Corporation, Germany). The macroscopic images of hydrogels were collected by using a digital camera and/or optical microscope (MVX10, Olympus, Shizuoka, Japan). Mechanical testing was executed on a universal testing machine (CMT6350, Shenzhen, China) with a tensile rate of 1 mm/min. The microstructural evaluation was performed via scanning electron microscopy (SEM; VEGA3 LMU, TESCAN, Tokyo, Japan).

## 3. Results and Discussion

### 3.1. One-Pot Anodic Electrodeposition of a Dual-Cation Crosslinked SA/CMC Hydrogel

**Figure 1:**
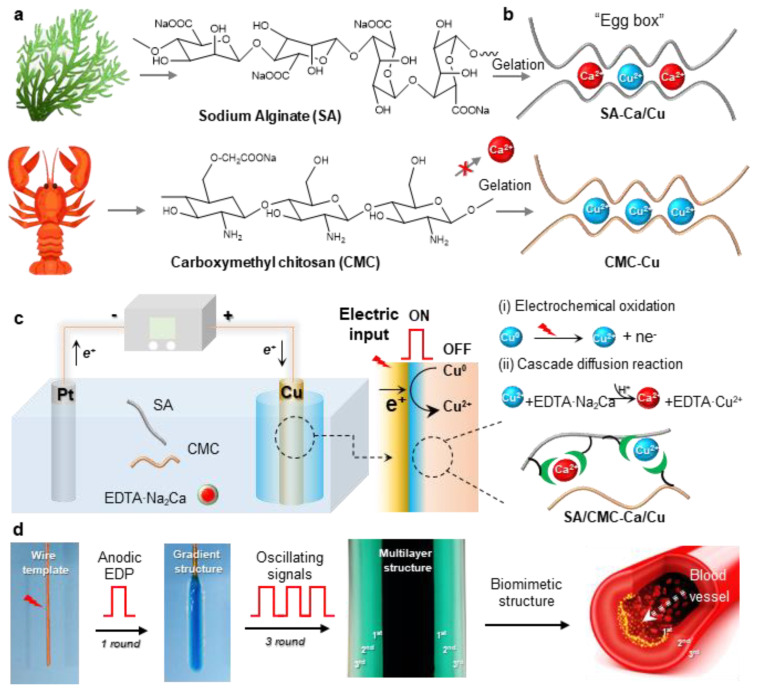
(**a**) Chemical structures of sodium alginate (SA) and carboxymethyl chitosan (CMC). (**b**) The two biopolymers show selective coordination interactions with metal cations (e.g., Ca^2+^ and Cu^2+^). (**c**) Schematically illustrates one-pot anodic electrodeposition of CMC/SA-Ca/Cu hybrid hydrogel and its cascade reaction mechanisms. (**d**) Using oscillating electrical signals to create vessel-like multilayer hydrogels.

Initially, this study aims to develop a biomimetic hydrogel that replicates the hierarchical architecture and tunable mechanical properties of human blood vessels. To ensure biological relevance and mechanical robustness, two naturally abundant hydrophilic biopolymers (alginate/SA, carboxymethyl chitosan/CMC) with ion-sensitive properties were selected to prepare an interpenetrating SA/CMC network (Figure 1a). As known from our previous reports, these polymers enable selective coordination with a pair of metal cations (e.g., Ca^2+^ and Cu^2+^) and exhibit distinct cation-binding gelation behaviors [32,33]: SA readily coordinates with Ca^2+^/Cu^2+^ via its “egg-box” molecular structure, while CMC shows weak interactions with Ca^2+^ (i.e., cannot induce gelation) but strong affinity for transition metals like Cu^2+^ (Figure 1b). Therefore, there is significant interest in developing dual-cation crosslinked SA/CMC hybrid hydrogels with tunable mechanical properties through controlled regulation of metal–polymer complex formulations.

To precisely tune the hydrogel structure, a one-pot anodic electrodeposition approach was employed for rapid fabrication of 3D hybrid hydrogels from sodium alginate/carboxymethyl cellulose (SA/CMC) blends. Conventional electrochemical oxidation methods typically rely on the partial dissolution of the anodic surface to generate metal cations that facilitate crosslinking via strong coordination with carboxylate groups in the SA/CMC matrix, resulting in a localized gelation at the electrode surface [34]. However, the rapid gelation kinetics and insufficient diffusion time for metal cations to penetrate beyond surface regions before triggering polymer chain sol–gel transition pose persistent challenges in constructing gas bubble-free 3D macroscopic hydrogel architectures [35]. To overcome these limitations, a pH-dependent competitive chelator such as calcium disodium ethylenediaminetetraacetate (EDTA·Na_2_Ca) was incorporated into the system to provide additional cation sources (e.g., Ca^2+^). The whole deposition process could be divided into two parts: (i) Electrochemical oxidation of the copper electrode generates Cu^2+^ ions, which subsequently coordinate with biopolymers to form an ultrathin yet mechanically robust hydrogel film featuring a well-defined interpenetrating polymer network architecture; (ii) concurrently, the water electrolysis generates localized acidic conditions, triggering EDTA·Na_2_Ca to release Ca^2+^ through competitive ligand exchange. These Ca^2+^ ions subsequently interact with SA to create a softer secondary network. The applied electric field directs cation migration, establishing a diffusion-driven gradient of Cu^2+^ and Ca^2+^ within the gel matrix. This cascade diffusion reaction process enables the spatial and rapid preparation of the hybrid hydrogel. Moreover, to replicate vessel morphology, a copper wire electrode template was integrated with pulsed electrical signals (cyclic ON–OFF model) (Figure 1d). The resulting free-standing hydrogel tube exhibits a hollow, multilayered architecture that mimics natural blood vessels, demonstrating high mechanical integrity for easy detachment from the template. Therefore, considering the multiple techniques and complex hydrogel structure, this one-pot anodic electrodeposition shows numerous advantages, such as being easy to control and environment-friendly and having high production efficiency and great controllability.

### 3.2. Hydrogel Growth and Surface Morphology

**Figure 2:**
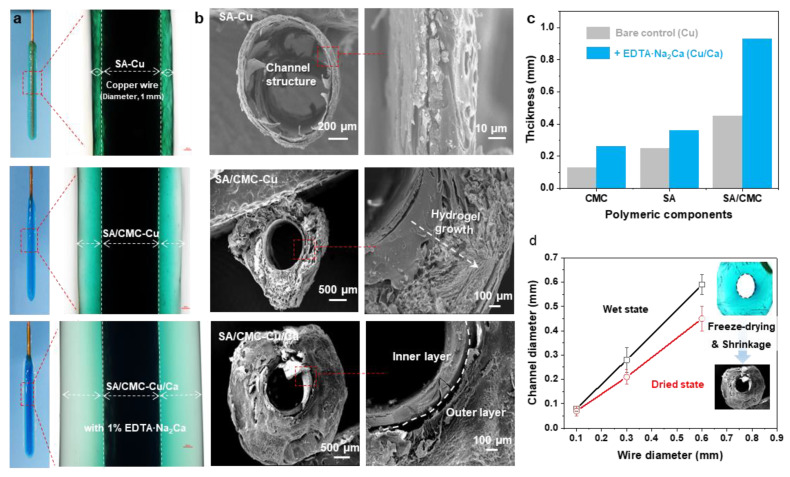
(**a**) The optical images of hydrogel coatings deposited on copper wire electrodes with different components (anodic current of 1.5 A/m^2^ for 180 s). (**b**) Cross-sectional SEM images of the hydrogel tube peeled off from the wire electrode. (**c**) Comparable study of hydrogel thickness prepared from various polymer and metal cation sources (anodic current of 1.5 A/m^2^, 120 s). (**d**) The channel structure was determined via the wire electrode template and freeze-drying treatment.

For comparison, the electrodeposition processes were carried out in SA, CMC, and SA/CMC solutions, respectively. As shown in Figure 2b, all the hydrogels could be generated on the wire electrode surfaces and displayed distinct color appearances. It can be seen that the SA and CMC hydrogels both show relatively higher values of thickness than other reports in such a dual cation system. These results could be attributed to the cascade diffusion reaction, enabling a rapid formation of the hybrid hydrogels. Interestingly, a much thicker hydrogel could be obtained from the SA/CMC mixture with different colors, providing primary evidence for the gradient structure generated within the gel matrix. These results suggested that the dual polymers could further improve the hydrogel growth efficiency and result in an obvious ion gradient in the hydrogel (i.e., Cu^2+^, blue to transparent). Next, to confirm the channel structure, the hydrogel samples were peeled off from the electrodes, rinsed with DI water, frozen in liquid nitrogen, and freeze-dried at −40 °C for 24 h. As shown in Figure 2b, the hydrogels both displayed a typical hollow structure with a similar diameter to the wire electrode, indicating that the tubular structure can be well-prepared via a wire electrode template. The CMC sample shows a compact and single-layer architecture that could be explained by its strong interactions formed between Cu but weak interactions with the Ca. The porous gradient could be observed from both the SA and SA/CMC. These phenomena are probably a result of the different metal–polymer coordination interaction between the dual cation and polymer chains during the gel forming process [36]. Considering the above ion-specific responsiveness, a dual-metal crosslinked tubular hydrogel was successfully designed with a gradient porous microstructure where Cu^2+^ primarily interacts with CMC to form a rigid primary network, while Ca^2+^ serves as a secondary crosslinker for SA. The hydrogel thicknesses were calculated from the optical images of each sample in the wet state using image analysis software (image J). After applying an anodic current of 1.5 A/m^2^ for 120 s, the hydrogel thicknesses were measured. As shown in Figure 2c, the addition of secondary metal cations (Ca^2+^) and an interpenetrating polymer network significantly improved the gelation rates, achieving a maximum improvement of 580% for the SA/CMC-Ca/Cu sample compared to the CMC-Cu sample prepared with a single polymer and metal crosslinker. The channel diameters of the free-standing tubular hydrogels measured at wet and freeze-dried states are displayed in Figure 2d. It can be seen that the channel diameters show a linear relationship with the wire electrode templates in the range of 0.06–0.58 mm. Moreover, the channel structure shows a slight shrinkage, suggesting the channel structure could be well controlled via the wire templates and further regulated via a freeze-drying treatment. These results indicate that achieving higher resolution is feasible through coupling this electrodeposition method with complementary approaches, such as capillary force-assisted assembly and/or condensed drying techniques.

**Figure 3:**
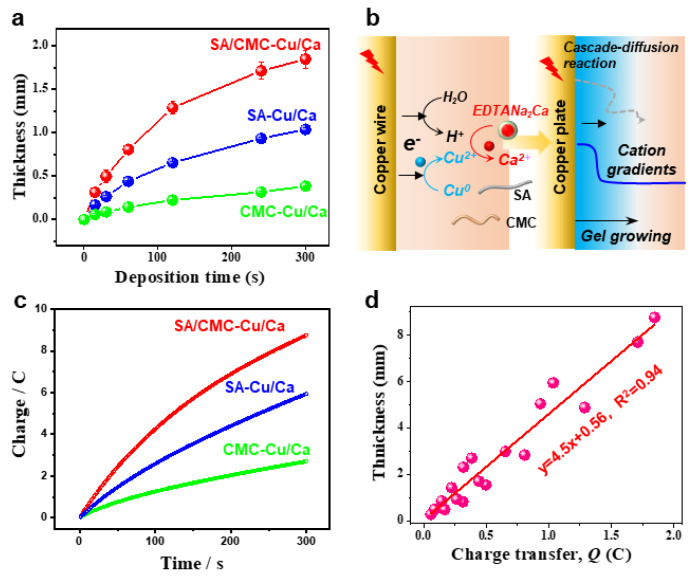
(**a**) Hydrogel thickness as a function of deposition time over 5 min (anodic current of 1.5 A/m^2^). (**b**) Schemes for the anodic electrodeposition and gel formulation. (**c**) The plots for charge as a function of deposition time over 5 min. (**d**) Quantification of the hydrogel thickness via transfer charges.

Next, we demonstrate that the anodic electrodeposition method has an excellent controllability and higher growth rates in the dual polymer solution containing a typical cation source. Figure 3a compares the hydrogels deposited on a copper wire over 300 s. The hydrogel formation kinetics exhibited a marked acceleration compared to control samples, as quantified by real-time thickness monitoring (Figure 3b). This enhancement is mechanistically attributed to two synergistic effects: (1) electric field-enhanced diffusion kinetics of metal ions and (2) preferential formation of dynamic coordination bonds between Cu^2+^/Ca^2+^ and carboxyl groups along the sodium alginate backbone. Moreover, the deposited hydrogel exhibits excellent thermodynamic stability due to strong coordination interactions between the biopolymers and metal ions. To systematically analyze the deposition dynamics, we established a linear correlation between cumulative charge transfer (Q) and deposition time (t), as shown in Figure 3c. The Q-t plot revealed a proportional relationship, with charge accumulation rates increasing upon metal cation supplementation. This trend confirms two critical processes: (i) progressive hydrogel formation through Faradaic reactions and (ii) interfacial charge transfer limitations caused by insulating hydrogel layers obstructing electrode–electrolyte contact. Figure 3d quantitatively correlates the hydrogel film thickness with cumulative charge transfer under electrochemical deposition conditions, systematically evaluating the influence of exogenous cation additives on interfacial ion migration kinetics. Interestingly, the linear relationships indicate a great controllability of the electrochemical gelation processes.

### 3.3. Metal–Polymer Coordination Interactions and Mechanical Properties

**Figure 4:**
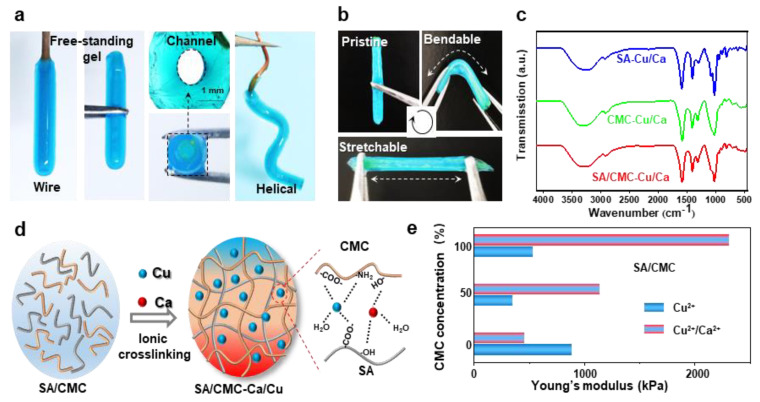
(**a**) Optical images show that the hydrogels could be easily peeled off and kept their hollow shapes; the gel could be prepared on a spiral-shaped wire electrode. (**b**) Optical images show that the hydrogels are mechanically strong and flexible. (**c**) FTIR. (**d**) Purposeful molecular interactions formed between the dual polymers and cations. (**e**) Mechanical properties of the hydrogels prepared with different components in dried states.

The optical images in Figure 4a demonstrate that tubular hydrogels can be successfully fabricated on various shaped electrodes (e.g., wire and helical). These hydrogel structures could be easily peeled off from the electrode surfaces while retaining channel architectures and similar dimensions comparable to those of their corresponding wire templates. The tubular hydrogels, fabricated through dual-cation crosslinking and interpenetrating network structure, exhibited exceptional mechanical strength and flexibility, enabling them to be repeatedly bent and stretched using tweezers without structural failure (Figure 4b). The internal molecular interactions were further revealed via the FT-IR measurements (Figure 4c). The Raman spectra of all specimens exhibited distinct vibrational signatures corresponding to their molecular architecture. In the pristine SA/CMC matrix, the characteristic bands at 3431cm^−1^ (O-H stretching of polysaccharide hydroxyl groups), 3260 cm^−1^ (N-H symmetric stretching in residual amino groups), 1590 cm^−1^, and 1416 cm^−1^ could be attributed to the stretching vibrations of asymmetric and symmetric carboxyl groups(-COO-), respectively [37]. Upon metal coordination, a red shift of -OH and -NH bands could be clearly observed, indicating the contributions of -NH_2_ and -OH groups in the metal–polymer complexation. Notably, the emergence of vibrational features at 620–610 cm^−1^ was ascribed to the stretching modes arising from chelation-induced charge transfer complexes. The absorption peaks of asymmetric and symmetric carboxyl groups show a blue shift behavior, suggesting that strong interactions are formed between carboxylic groups and metal ions [38]. The proposed molecular interactions for mechanical enhancements are schematically illustrated in Figure 4d. Specifically, the CMC/SA-Cu complexations maintain hydrogel network structural integrity, while the dynamic selective SA-Ca ionic bonds network dissipates energy during deformation, collectively contributing to the observed mechanical robustness. These ions could strongly coordinate with the active carboxylate (-COO^−^) and hydroxyl (-OH) groups from SA and CMC chains, forming ionic crosslinks and an interpenetrating network in the hydrogel polymer matrix. Overall, it could be inferred that the carboxylic, -OH, and -NH_2_ groups were all involved in the fast complexation of metal–polymer complexations. The tunable mechanical properties of the hybrid hydrogels were tested in Figure 4e. By tuning the ratios of biopolymers and ions, the synergetic interplay between the interpenetrating polymeric networks and coordination systems enabled precise modulation of hydrogel stiffness. To further investigate mechanical properties, all hydrogels underwent re-equilibration prior to cyclic mechanical testing at physiological temperature (37 °C). The elasticity of the rehydrated hydrogel was evaluated with the corresponding data presented in Figures S1 and S2. Following cyclic stretching, both hydrogel formulations demonstrated robust mechanical performance. Notably, the dual-cation crosslinked hybrids exhibited higher strength and lower energy dissipation compared to single-cation systems, maintaining superior mechanical integrity post-cycling. Both hydrogel samples showed an initial strength reduction followed by stabilization during cyclic loading. This deformation behavior indicates strong structural integrity retention and durability under simulated bio-thermomechanical conditions.

### 3.4. Creating Multilayer Structure in the Hydrogel by Using Pulsed Electrical Signals

**Figure 5:**
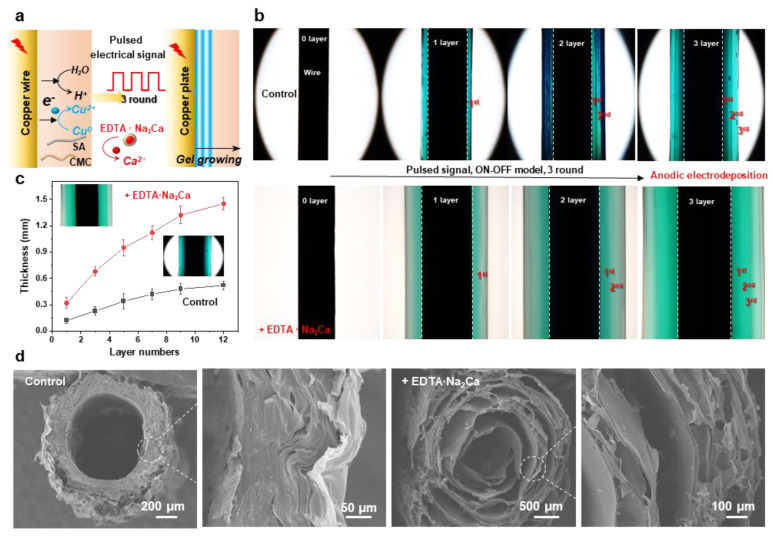
(**a**) Electrodeposition of the hybrid hydrogel with tunable hierarchical structure by using an oscillating electric signal. (**b**) Optical images of the multilayered hydrogels prepared with or without 1% EDTA·Na_2_Ca; the typical 3-layer samples were carried out by processing 3 cycles of ON step (1.5 A/m^2^, 60 s) and OFF step (0 A/m^2^, 5 s). (**c**) Hydrogel thickness as a function of layer numbers (1.5 A/m^2^, 30 s, 1~12 rounds). (**d**) Cross-sectional SEM images show the multilayer internal structures of the hydrogels.

As an emerging coating methodology, cathodic electrodeposition of biopolymers has captured significant attention in bio-related fields due to its environmentally friendly synthesis conditions, low operational voltage, ease of operation, and precise controllability [39,40]. However, developing a versatile method to construct polymer coatings with tunable structures and functionalities remains a critical research objective. In our previous work, multilayer hydrogel films with adjustable layer numbers and thicknesses can be fabricated through spatiotemporally controlled electrodeposition using pulsed electrical signals. Therefore, in this section, we extend pulsed cathodic electrodeposition to a one-pot anodic system employing programmed electrical signals (Figure 5a). The pulsed electrical signals (ON–OFF model) operated as follows: during the ON step (1.5 A/m^2^ anodic current for 60 s), electrical stimulation triggered molecular self-assembly and localized gelation, while the OFF step (0 A/m^2^ for 5 s) induced a hydrogel growth interruption. The multilayer hydrogels deposited with/without the addition of EDTA·Na_2_Ca on copper wire electrodes are compared in Figure 5b. Notably, constant total charge consumption (Q =i*t) was maintained across all cycling deposition experiments to ensure consistent film gelation during the ON step. It can be seen that both hydrogels exhibited a three-layer internal structure, and a higher gelation rate can be observed from the sample prepared with the integration of EDTA·Na_2_Ca. Hydrogel thickness showed a positive correlation with layer numbers, which could be precisely controlled via ON–OFF cycle counts (Figure 5c). Finally, the microstructures were further tested by the SEM, and the results indicated that the multilayer architecture was still maintained in the freeze-dried samples. The layer number and thickness could be easily adjusted via the electrical signal sequences, while the channel structure was determined via the wire templates (Figure 5d). Finally, this study establishes a field-assisted additive manufacturing strategy where electrical signals dynamically control hydrogel growth (quantified by charge transfer) while intermittent signal interruptions enable microstructure engineering

### 3.5. Antibacterial Properties

**Figure 6:**
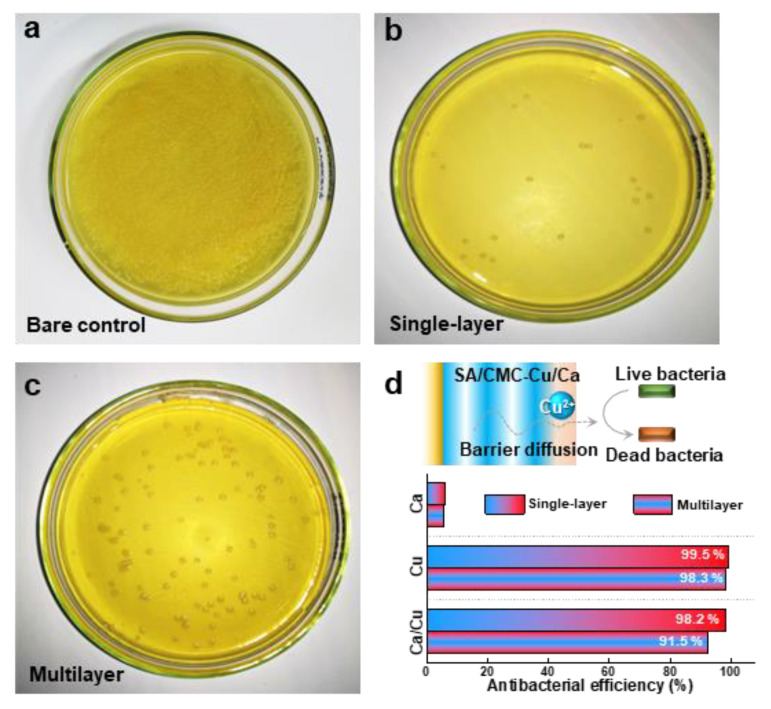
Antibacterial activities of the multilayer structured hydrogels (SA/CMC-Ca/Cu) against *S. aureus* determined by bacterial counts: (**a**) bare control, (**b**) single-layer, and (**c**) multilayer. (**d**) Schematic diagram and antibacterial efficiency illustrating the barrier diffusion principle in metal-based multilayer hydrogel-induced antibacterial performance.

In the final section, we compared the antibacterial properties of the hybrid hydrogels prepared with different internal structures. Figure 6a is provided as the control that was prepared without hydrogel co-culture treatment. The SA/CMC-Cu/Ca hybrid hydrogel prepared with single-layer and multilayer structures both showed excellent contact antibacterial activity (Figure 6b,c). These results agree well with the great antibacterial activities of the entrapped metal cations (i.e., Cu^2+^), which progressively diffuse out from the hydrogel network and kill the bacteria [41]. The hydrogels loaded with a single metal ion have also been tested. The results indicate that Ca^2+^ shows little antibacterial activity, while copper exhibits excellent antibacterial performance. These results are consistent with other reports, and the observed antibacterial properties can be attributed to the diffusion of metal ions that contact and subsequently kill the bacteria. Interestingly, the multilayer hydrogel shows a lower antibacterial efficiency (91.5%) as compared to that of the single-layer sample (98.2%). This phenomenon indicates a barrier diffusion process of copper ions, processed and released from the multilayered hydrogel (Figure 6d). The experimental data paradoxically demonstrate that controlled ion release kinetics significantly enhance antimicrobial efficacy. The reformulated hydrogel system exhibits great pH-responsive stability while enabling sustained copper ion release that aligns with optimal wound healing parameters. Moreover, the cytotoxicity of the deposited hydrogels was evaluated using an MTT assay with NIH3T3 cells (Figure S3). Both the Ca^2+^- and Cu^2+^-containing hydrogels exhibit low cytotoxicity to NIH3T3 cells, supporting their potential for biomedical applications. Coupling low cytotoxicity with tunable antibacterial functionality through electrodeposition-controlled material architectures suggests great potential applications in skin repair and wound healing fields, especially in preventing bacterial inflammation.

## 4. Conclusions

In summary, a novel one-pot anodic electrodeposition strategy for fabricating biomimetic hybrid hydrogels with hierarchical architectures and excellent antibacterial properties has been successfully developed. By integrating dual metal cations (Ca^2+^/Cu^2+^) and interpenetrating biopolymer networks (alginate/CMC), we achieved spatially controlled gelation through cascade coordination reactions and electric field-directed ion diffusion. This methodology overcomes traditional limitations in 3D hydrogel fabrication by combining EDTA·Na_2_Ca-mediated Ca^2+^ release with pulsed electrical signals, enabling precise control over layer formation and gradient microstructures. Experimental results revealed that the multilayer hydrogels exhibited moderate copper ion release (91.5% efficiency) compared to single-layer counterparts (98.2%), attributed to barrier diffusion effects. The hydrogel’s hollow tubular morphology, replicating vascular dimensions (0.06–0.58 mm channel diameter), was maintained through freeze-drying with minimal shrinkage (<5%). This electrodeposition platform not only establishes a scalable route to engineer organ-mimetic hydrogels but also introduces a broader paradigm for transducing electrical signals into biological architectures, bridging the information-processing capabilities of electronics with the functional demands of vessel tissue engineering. By integrating electrode templating, pulsed crosslinking, and reaction-diffusion principles, our work opens new avenues for developing next-generation vessel grafts with patient-specific mechanical and biochemical functionalities.

## Data Availability

The original contributions presented in this study are included in this article. Further inquiries can be directed to the corresponding author.

## Supplementary Materials

The following supporting information can be downloaded at: https://www.mdpi.com/article/10.3390/jfb16070235/s1, Figure S1: Mechanical properties of the SA/CMC-Cu and SA/CMC-Cu/Ca rehydrated hydrogel films after being stretched with 3 cycles; the rehydrated samples were prepared by drying the hydrogel sample at 45 ℃ for 6 h and then re-swelled in water at room temperature for 24 h; Figure S2: Comparable studies of the mechanical properties of SA/CMC-Cu and SA/CMC-Cu/Ca rehydrated hydrogel films after being stretched with different cycles; Figure S3: The cell cytotoxicity was determined from the viabilities of NIH3T3 cells co-cultured with different concentrations of hydrogel leaching solutions (48 h); prior to measurements, all hydrogels underwent thorough rinsing with deionized water.
