# Supplementary material for: One-Pot Anodic Electrodeposition of Dual-Cation-Crosslinked Sodium Alginate/Carboxymethyl Chitosan Interpenetrating Hydrogel with Vessel-Mimetic Heterostructures
Source: J Funct Biomater. 2025 Jun 26;16(7):235. doi: 10.3390/jfb16070235 (PMC12294871; doi:10.3390/jfb16070235)
Supplement: Supplementary file 1 [file jfb-16-00235-s001.zip › jfb-3694942-supplementary.pdf]

Supporting Information

For

One-Pot Anodic Electrodeposition of Dual-Cation-Crosslinked Sodium Alginate/Carboxymethyl Chitosan Interpenetrating Hydrogel with Vessel-Mimetic Heterostructures

Xuli Li[1], Yeqing Qu[2], Yong Zhang[1], Pei Chen[1], Siyu Ding[1], Miaomiao Nie[1], Kun Yan*[1], Shefeng Li*[2]

[1]Hubei Province Key Laboratory of Agricultural Waste Resource Utilization, School of Chemistry and Environmental Engineering, Wuhan Polytechnic University, Wuhan 430023, China

[2]Key Laboratory of Textile Fiber & Product, Ministry of Education, Wuhan Textile University, Wuhan 430200, China

*Corresponding Author: kyan@wtu.edu.cn; lishengfeng@whpu.edu.cn;

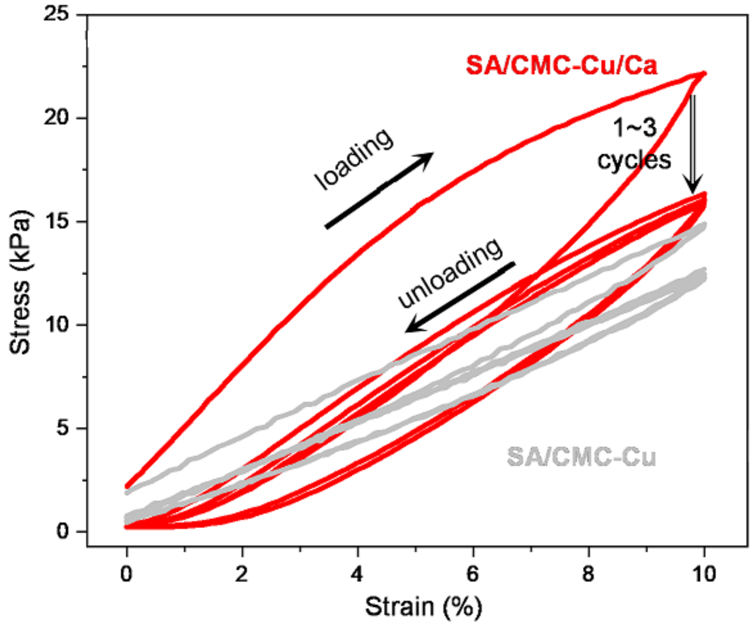

Figure S1. Mechanical properties of the SA/CMC-Cu and SA/CMC-Cu/Ca rehydrated hydrogel films after being stretched with 3 cycles; the rehydrated samples were prepared by drying the hydrogel sample at 45 °C for 6 h and then re-swelled in water at room temperature for 24 h.

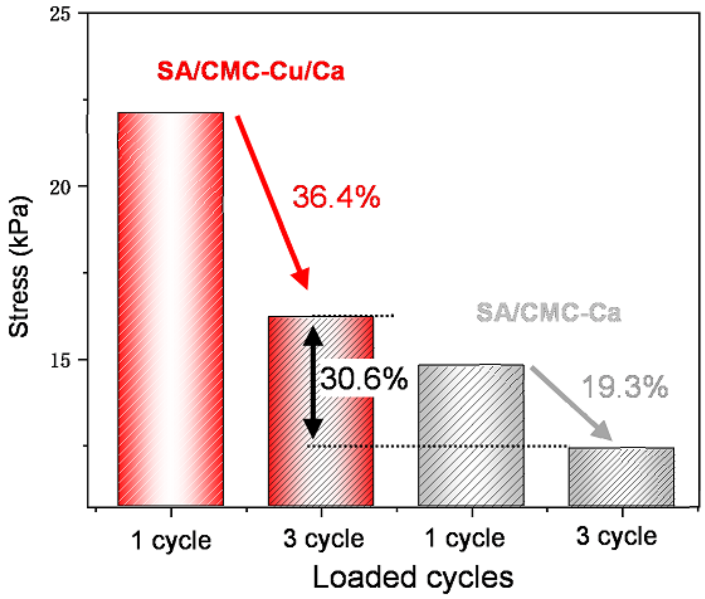

Figure S2. Comparable studies of the mechanical properties of SA/CMC-Cu and SA/CMC-Cu/Ca rehydrated hydrogel films after being stretched with different cycles.

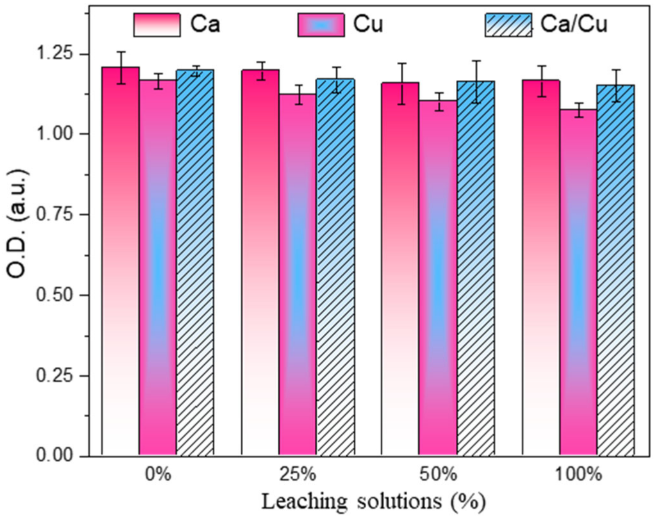

Figure S3. The cell cytotoxicity was determined from the viabilities of NIH3T3 cells co-cultured with different concentrations of hydrogel leaching solutions (48 h); prior to measurements, all hydrogels underwent thorough rinsing with deionized water.

Cell cytotoxicity:

The cytotoxicity of the deposited hydrogels was evaluated using an MTT assay with NIH3T3 cells. The leaching solutions at varying concentrations collected from each sample were co-cultured with the cells for 48 h. The final cell viability was determined by optical density (O.D.), and with cells cultured

without leaching solution (0 %) serving as controls. The Ca^{2+}-containing hydrogels exhibited consistently high O.D. values across all leaching concentrations, indicating low cytotoxicity towards NIH3T3 cells (Figure S3). Similarly, Cu^{2+}-incorporated samples showed O.D. values comparable to the Ca^{2+} controls. This demonstrates minimal impact on cell growth and acceptable biocompatibility, consistent with existing literature. Furthermore, the samples maintain viability levels similar to both hydrogel samples tested with different concentrations. These results indicate that both Ca^{2+} and Cu^{2+}-containing hydrogels exhibit low cytotoxicity to NIH3T3 cells, supporting their potential for biomedical applications.
